# Spotlight on early-career researchers: an interview with Toshiro Moroishi

**DOI:** 10.1038/s42003-019-0581-5

**Published:** 2019-09-13

## Abstract

Toshiro Moroishi is an Associate Professor at the Department of Molecular Enzymology at Kumamoto University, Japan where he has led his own group since December 2017. Research in his lab is focused on the role and regulation of Hippo signalling in development and cancer with a specific interest in the role for Hippo in immunosuppression. He tells us about his research interests, career and lessons learned along the way, as part of our series on early-career researchers.

**Figure:**
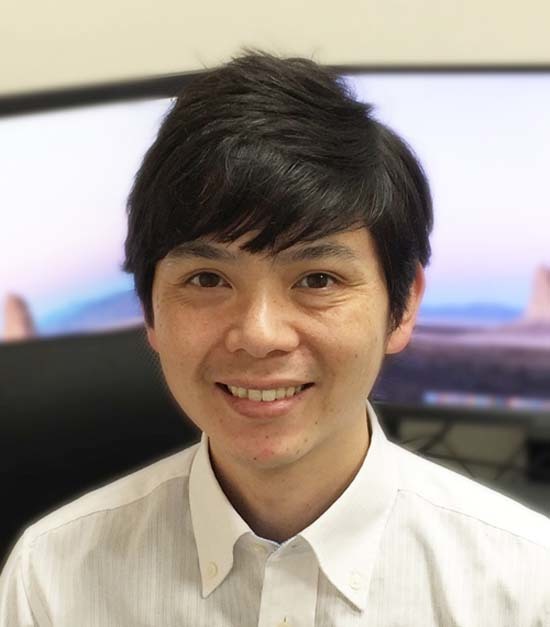
Toshiro Moroishi

Please tell us about your research interests

In multicellular organisms like human, different types of differentiated cells are cooperatively organized to form tissues and organs in the body. My long-term research interests involve physiological mechanisms regulating tissue homeostasis and integrity. I would like to understand how cells sense their environment and appropriately adapt their biological activities, especially in terms of cell proliferation and differentiation. Cells continuously detect broad signals from their environment and integrate the information to decide their own behaviours. The Hippo intracellular signalling pathway, initially identified through fly genetics, responds to a wide range of physiological cues from the environment, including extracellular growth signals, mechanical signals, nutrition, and stress signals. The Hippo pathway thus acts as a platform for multiple signals, contributing to cell fate determination. Currently, our research team is focusing on the molecular and cellular regulation of and by the Hippo pathway. The goal of our research is to understand molecular mechanisms operating in tissue physiology as well as pathological disruptions, such as tissue degeneration and cancer. We aim to provide scientific basis for drug discovery for those diseases.

Why did you choose to be a scientist?

I entered medical school to become a clinical doctor in 2002. Actually, I never imagined that I would be a basic scientist until the last year of medical school. When I started a clinical clerkship during the final two years of study, I realized that there are still many diseases that we cannot do much about. In particular, treatment for advanced stages of cancer is very challenging with limited therapeutic options. I also learned that a variety of current medications and procedures have been discovered in efforts to control diseases by previous research, and continuous research and development efforts will shape the future of medicine. At that time, and even now, Japan has a serious concern that the number of basic scientists who learned clinical medicine is continuously decreasing, which makes it difficult to keep and improve scientific productivity. Therefore, I decided to enter graduate school and learn more about science. Right after graduation from medical school in 2008, I entered graduate school and started my scientific career in molecular biology and biochemistry. My thesis work investigates the molecular mechanisms that achieve and maintain the homeostatic control of mammalian iron metabolism in embryogenesis and adult tissue physiology, and I earned a Ph.D. in Medical Sciences in 2012. I really enjoyed being a basic researcher during my Ph.D. training, and therefore, I decided to keep on working as a basic scientist.

What are your predictions for your field in the near future?

We have recently revealed that the Hippo pathway in cells affects the induction of a host immune response. The reciprocal dialogue between cells and the immune system is pivotal to ensure tissue homeostasis by eliminating undesirable cells and orchestrating tissue integrity. Indeed, recent advances in cancer immunotherapy have provided new therapeutic approaches for the advanced stage of cancer, and several immune checkpoint inhibitors show remarkable therapeutic outcome in the clinic. The treatment options for patients with cancer have thus changed considerably over the last decade. Although the current immunotherapy approach still has several problems, such as low response rate and severe side effects, continuous research efforts will likely ensure a successful therapeutic approach for cancer. Therefore, it would be important to further elucidate the molecular mechanisms of the reciprocal interaction between cells and the immune system in the context of tissue homeostasis, and to uncover the physiological and pathological functions of tissue editing by the immune system. Further investigations delineating the mechanistic link between the Hippo pathway and immune response will have important implications in understanding pathological disruptions of tissue homeostasis, such as cancer.

Can you speak of any challenges that you have overcome?

Being an independent researcher is always challenging. After 4 years of postdoctoral research in the United States, I started looking for a principal investigator (PI) job in late 2016. I sought an independent position both in Japan and the United States, but there are very few PI positions open for researchers in Japan, which made it difficult to find a job. I applied to more than 10 institutes and got several interviews and finally decided to accept the current position in late 2017. Soon after I started my own lab, the next issues standing in the way of my scientific career were (1) getting research grants, and (2) recruiting highly motivated students and postdocs. I applied for more than 20 grants (essentially everything that I was eligible to apply for) and managed to secure enough funds to set up the lab equipment. Setting up your own research team is challenging, but I was able to recruit motivated researchers by giving scientific seminars, providing information through the lab webpage, and getting advice and help from the other senior PIs. It took about a year to settle down to focus on the research. I learned that working alone is not sufficient and getting help from kind colleagues is pivotal when starting a new lab.

What advice would you give to your younger self?

I would like to say “believe yourself and simply enjoy your science”. When I started my own scientific career 11 years ago, I was anxious and jittery about my future career development. I was concerned that I may not be talented enough to survive the scientific competition and someday I may lose my job. However, I learned that no one is talented enough not to have to work hard, and I can always hope that any efforts will be rewarded in the future. As long as I keep on running, there is always a way ahead of me no matter how difficult the situation may be. It is important to be optimistic and to focus on the science in front of me. The five points that I believe are important for scientific career development are (1) to love and enjoy your science, (2) to clarify your vision and mission, (3) to have clear strategy, (4) to communicate with colleagues, and (5) to work hard. I hope to keep on developing my own science by always keeping those points in mind.

What is the funniest or most memorable thing that has happened to you in the lab?

There are two memorable events from my previous research experience. The first one was discovering that the embryonic lethality of iron-overload mice deficient in iron-sensing ubiquitin ligase FBXL5 could be rescued by additional ablation of its substrate IRP2^1^. I was impressed by the beauty of genetic approach in studying physiological relationship between two molecules. The second memorable thing is that inhibition of the Hippo pathway in cancer cells exerts completely opposite effects on cancer cell growth in vitro and in vivo^2^. Deletion of the Hippo pathway kinases LATS1/2 caused a moderate increase of anchorage-independent growth of cancer cells in vitro but induced strong immune responses and led to inhibition of tumour growth in immune-competent mice. I was surprised by the complexity of multicellular organisms where different types of cells cooperatively maintain tissue homeostasis and thus show completely unexpected outcome on tumour growth. Every time I find something new, I am impressed by the complex and ingenious life system that the human being employs, and it may be the reason why studying science is fantastic for me.

*This interview was conducted by Senior Editor Christina Karlsson Rosenthal*.
